# Metabolomic and Lipidomic Tools for Tracing Fish Escapes from Aquaculture Facilities

**DOI:** 10.1021/acsfoodscitech.3c00589

**Published:** 2024-03-21

**Authors:** Warda Badaoui, Frutos C. Marhuenda-Egea, Juan Manuel Valero-Rodriguez, Pablo Sanchez-Jerez, Pablo Arechavala-Lopez, Kilian Toledo-Guedes

**Affiliations:** † Department of Biochemistry and Molecular Biology and Agricultural Chemistry and Edafology, University of Alicante, Carretera San Vicente del Raspeig s/n, 03690 Alicante, Spain; ‡ Department of Biological Sciences, University of Bergen, Postboks, 7803 5020 Bergen, Norway; § Department of Marine Sciences and Applied Biology, University of Alicante, Carretera San Vicente del Raspeig s/n, 03690 Alicante, Spain; ∥ Mediterranean Institute of Advanced Studies (IMEDEA-CSIC), C/Miquel Marquès 21, 07190 Esporles, Spain

**Keywords:** metabolomics, lipidomics, NMR, marine aquaculture, fish escape events, fish traceability

## Abstract

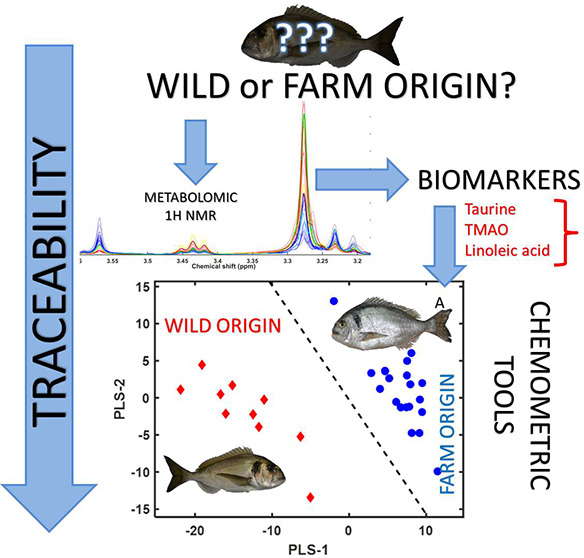

During adverse atmospheric events, enormous damage can occur at marine aquaculture facilities, as was the case during Storm Gloria in the southeastern Spanish Mediterranean in January 2020, with massive fish escapes. Fishes that escape were caught by professional fishermen. The objective of this study was to identify biomarkers in fish that enable differentiation among wild fish, escaped farm-raised fish, and farm-raised fish kept in aquaculture facilities until their slaughter. We focused on gilthead sea bream (*Sparus aurata*). We used nuclear magnetic resonance to search for possible biomarkers. We found that wild gilthead sea bream showed higher levels of taurine and trimethylamine-N-oxide (TMAO) in their muscle and higher levels of ω-3 fatty acids, whereas farm-escaped and farmed gilthead sea bream raised until slaughter exhibit higher levels of ω-6 fatty acids. From choline, carnitine, creatinine, betaine, or lecithin, trimethylamine (TMA) is synthesized in the intestine by the action of bacterial microflora. In the liver, TMA is oxidized to TMAO and transported to muscle cells. The identified biomarkers will improve the traceability of gilthead sea bream by distinguishing wild specimens from those raised in aquaculture.

## 1 Introduction

Aquaculture animal production reached 87.5 million tons (worth USD 264.8 billion) in 2022,^1^ and production has continued to grow in recent years. Caged-based marine fish aquaculture involves raising fish in open-water enclosures, such as cages, pens, and net pens, in saltwater or brackish water. The practice expanded in Europe, Japan, and the United States in the 1960s and 1970s and grew rapidly in Asia, particularly in China, Taiwan, and Indonesia, in the 1980s.^2^ Presently, cage-based aquaculture is an important contributor to global aquaculture production, offering several advantages over traditional aquaculture practices, such as the ability to raise fish in their natural environment on a large scale. The cages used in aquaculture can be constructed from various materials, including plastic and metal, and can be designed to meet the environmental conditions required for the species to be raised. However, the escape of farmed fish from sea cages is considered a major environmental issue in marine aquaculture and is seen as a threat to marine biodiversity.^3^

Escaped fish can have negative ecological consequences on native populations due to interbreeding, competition for food and/or habitats, and transmission of diseases to wild fish and other farmed stocks.^4^ The escape of farmed fish from marine aquaculture facilities is common, even in systems linked to natural settings.^5−7^ Indeed, between 2007 and 2009, approximately 9 million farmed fish escaped from sea-cage fish farms in European marine aquaculture facilities.^8^ Severe storms can also lead to mass escapes, affecting marine finfish aquaculture sectors across the globe.

Artisanal fishermen have been known to capture escaped farmed fish and sell them alongside wild fish.^5,7,9^ Metabolomics and lipidomics are techniques used to measure small molecules (polar and apolar) from tissues or biofluids and generate metabolic profiles.^10−12^ These profiles can be used to detect alterations caused by environmental factors, pollutants, or other factors.^13,14^ These techniques are now applicable to various domains such as disease diagnostics, toxicology, plant science, and nutrition,^10^ and analytical instruments for the measurement of metabolites are continuously under development.^15^ Extracting metabolites from tissues is considered one of the key points in metabolomics studies.^16^ However, ^1^H nuclear magnetic resonance (NMR) spectroscopy and mass spectrometry are commonly used analytical methods in metabolomics owing to their high sample throughput and automated analysis capabilities.^17^ NMR is not only widely used in metabolomics but also for structural analysis in proteomics and lipidomics, and NMR spectroscopy can rapidly generate a large amount of spectrum data.^18^ Multivariate pattern recognition methods, such as principal component analysis, partial least-squares discriminant analysis, and orthogonal partial least-squares discriminant analysis, are often used to minimize the dimensionality of data,^19^ screen main metabolites, and distinguish between different groups. These techniques have broad applications, including food nutrition evaluation and food function interpretation.^20^

The gilthead sea bream (*Sparus aurata*), an important aquaculture species in the Mediterranean (Fazio et al.), has been the subject of concern related to traceability, labeling, and fraud, owing to the intensive production^21^ and low fisheries output of the species. Therefore, in this study, the potential use of metabolic and lipidic biomarkers to distinguish between wild gilthead sea bream and their farm-escaped counterparts was evaluated. To this end, the muscle composition and fat deposition of gilthead sea bream purchased from fish markets (wild and potential farm escapees) and aquaculture facilities on the Spanish Mediterranean coast were analyzed.

## 2 Materials and Methods

### 2.1 Specimen Collection and Sample Preparation

In total, 30 gilthead sea breams were collected from fish markets, supermarkets, and local and wholesale markets in the Valencian Community and Murcia (Spain) in 2019–2022. Before the metabolomic and lipidomic analyses were performed, the external state of individual fish was examined for parasites, and biometric measurements and photographs were taken. Based on their appearance, the presence of regenerated scales^9^ and traceability references (“commercial” labeling), the fish were classified into the following origin groups: “Wild”, “Escape”, and “Cultured” (*n* = 10).

Following classification, a portion of fish muscle was extracted and frozen with liquid nitrogen, after which it was ground into a fine powder. This pulverized muscle was extracted using the Bligh–Dyer method,^22^ and the resulting polar and nonpolar fractions were dried using a SpeedVac. The metabolites were then suspended in either 500 μL of H_2_O [containing 50 μL of D_2_O with 0.75% 3-(trimethylsilyl) propionic-2,2,3,3-d4 acid sodium salt (TSP) and 0.1% sodium azide] or CDCl_3_, depending on their polarity.

### 2.2 ^1^H NMR Acquisition and Data Processing Parameters

A 500 μL sample was placed in a 5 mm NMR tube, and spectra were referenced to TSP at 0.00 ppm (polar samples) or to chloroform at 7.26 ppm. All ^1^H NMR experiments were performed on a Bruker Avance 400 MHz equipped with a 5 mm HBB13C TBI probe with an actively shielded Z-gradient. The 1D solution state ^1^H NMR experiments had a 2 s recycle delay, 32,768 time-domain points, and 2.556 s acquisition time. In total, 1024 scans were performed, and the experiment was conducted at 298 K. Spectra were apodized through multiplication with an exponential decay, producing a 0.3 Hz line broadening in the transformed spectrum. The ^1^H NMR spectra were normalized and reduced to ASCII files using TopSpin (Bruker) and aligned using icoshift (version 1.0; available at www.models.kvl.dk).^23^ Processing of ^1^H NMR spectra was performed in MATLAB (MathWorks, Natick, MA, USA). The region of water (4.60–4.95 ppm) and extreme high and low fields (<0.5 and 10 ppm, respectively) were removed. Metabolites were identified in one-dimensional spectra using The Human Metabolome Database (HMDB, https://hmdb.ca/) and the literature cited in this study.

### 2.3 Statistical Analysis

The NMR data were imported into MATLAB. The TIC data profiles were organized into a matrix and analyzed using the PLS-LDA algorithm,^24^ a supervised method that groups data according to a mathematical model. This algorithm allows for determining whether data are grouped correctly and which properties are important for accurate classification. The key statistical parameters used to determine the accuracy of the model are R2Y, R2X, specificity, sensitivity, and AUC. We used pareto in the data analysis with PLS-LDA, applying three components.^24^

### 2.4 Data Availability

Raw spectroscopical data were deposited at Mendeley Data: Marhuenda, Frutos (2023), ‘*Sparus aurata* H NMR spectra’, Mendeley Data, V1, doi: 10.17632/rxykc8bw2h.1.

## 3 Results

**Figure 1:**
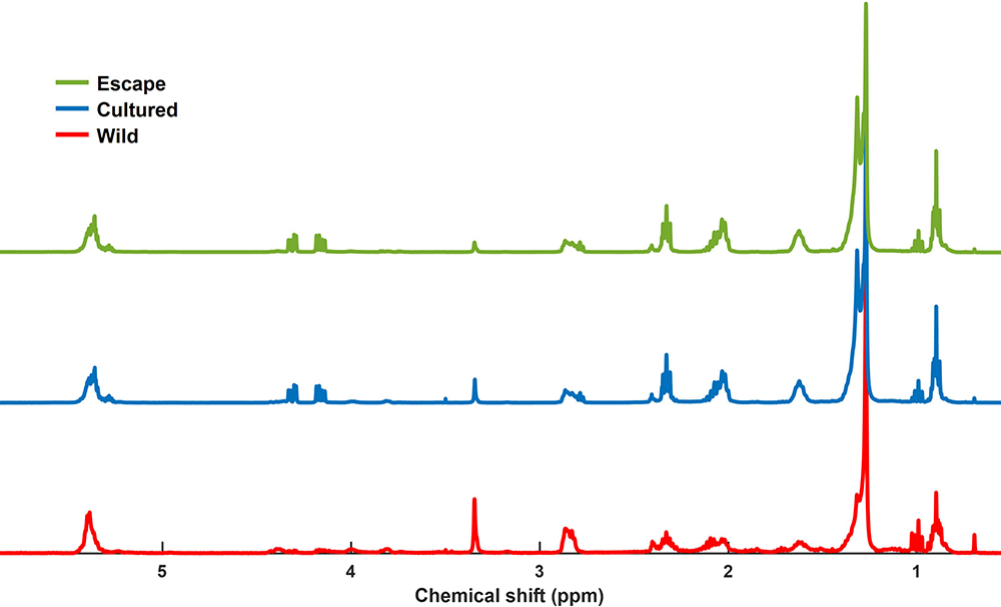
Representative ^1^H NMR spectra of the polar fractions of gilthead sea bream (*Sparus aurata*) muscle samples.

The samples extracted from gilthead sea bream muscles were analyzed via ^1^H NMR, and the relevant peaks in the polar phase ^1^H NMR spectra were identified through comparisons with relevant literature^25−28^ and a metabolite database (HMDB; https://hmdb.ca/) (Table 1). Some spectra are shown in Figure 1.

**Table 1:** Polar Signal Assignments for ^1^H NMR Spectra of Gilthead Sea Bream (*Sparus aurata*) Muscle Samples^a^

chemical shift (ppm)	multiplicity	group	compound
0.94	t	δ-CH3	isoleucine (Ile)
0.97	d	δ-CH3/δ′-CH3	leucine (Leu)
0.99	d	γ-CH3	valine (Val)
1.02	d	γ-CH3	isoleucine (Ile)
1.05	dd	γ′*-*CH3	valine (Val)
1.19	t	CH3	ethanol
1.33	d	γ-CH3	threonine (Thr)
1.34	d	–CH3	lactate
1.48	d	β-CH3	alanine (Ala)
1.71	m	δ-CH2	lysine (Lys)
1.72	m	γ-CH	leucine (Leu)
1.93	s	CH3	acetic acid (AA)
2.13	s	S-CH3	methionine (Met)
2.14	m	β,β′-CH2	glutamine (Gln)
3.04	s	N–CH3	creatine (Cr)/phosphocreatine (PCr)
3.23	m	α-CH2 (β-Ala)	anserine (Ans)
3.27	s	N–CH3	TMAO
3.42	t	N–CH2	taurine (Tau)
3.56	m	CH-2 (Glu)	sucrose (Suc)
3.68	d	α-CH	isoleucine (Ile)
3.90	dd	α-CH	aspartic acid (Asp)
3.93	s	α-CH2	creatine (Cr)/phosphocreatine (PCr)
4.13	q	–CH	lactate
4.12	q	α-CH	lactic acid (La)
6.11	d	CH-1 (Rib)	inosine (Ino)
6.74	s	–CH=CH–	fumarate
7.19	s	CH-2,6	tyrosine (Tyr)
8.23	s	C2H	histidine (in anserine)
8.35	s	CH	formate
8.52	s		unassigned

a Abbreviations: s, singlet; d, doublet; t, triplet; m, multiplet; and dd, doublet of doublets.

**Figure 2:**
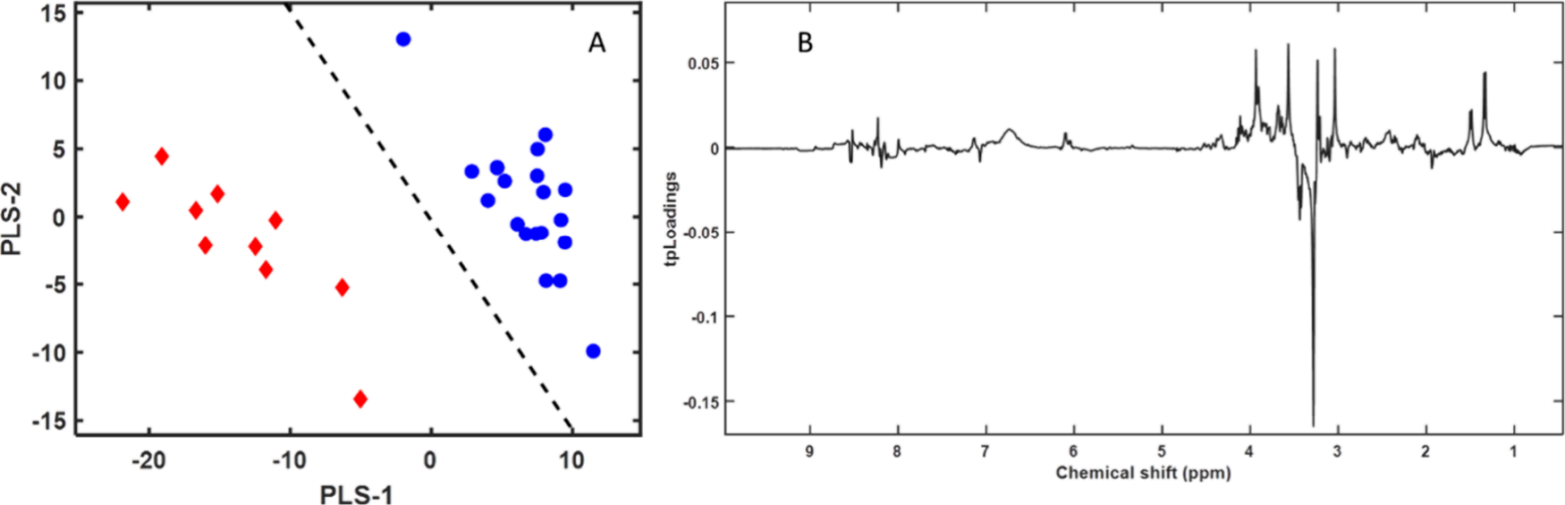
(A) First two components of the PLS-LDA model score plots of ^1^H NMR spectra for the polar fraction of gilthead sea bream (*Sparus aurata*) muscle samples: wild fish (red diamonds) and farm-raised and farm-escaped fish (blue circles). (B) Pseudospectrum formatted PLS-LDA tpLoading. Peak intensity (positive or negative) in the pseudospectrum represents the most significant spectral shift regions in the PLS-LDA model. The cumulative R2Y and R2X values for the three variables were 0.88 and 0.50, respectively. The error was 0, the sensitivity was 1, the specificity was 1, and the AUC was 1.

Having identified the metabolites in the polar fraction of the gilthead sea bream muscle tissue, we performed chemometric analysis of the spectra by constructing classification models using PLS-LDA^24^ to group the gilthead sea bream samples into three categories: wild, farm-escaped, and farm-raised until slaughter. PLS-LDA is a supervised method that identifies the most important spectrum signals for model building,^24^ allowing for separation of the fish groups and improving their traceability. We evaluated model quality using statistical parameters such as R2X, R2Y, sensitivity, specificity, and AUC. In the first model, we compared wild fish to a group formed by the other two categories (farm-escaped and farm-raised until slaughter) (Figure 2).

**Figure 3:**
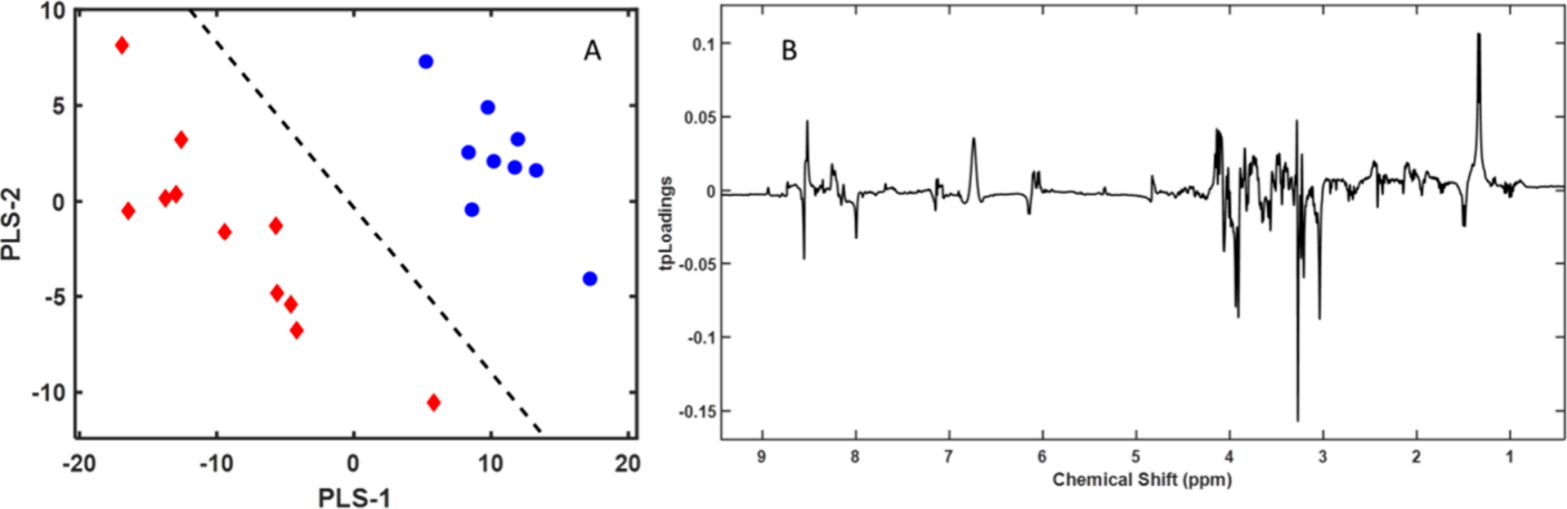
(A) First two components of the PLS-LDA model score plots of ^1^H NMR spectra for the polar fraction of gilthead sea bream (*Sparus aurata*) muscle samples: farm-escaped fish (red diamonds) and farm-raised fish (blue circles). (B) Pseudospectrum format PLS-LDA tpLoading. Peak intensity (positive or negative) in the pseudospectrum represents the most significant spectral shift regions in the PLS-LDA model. The cumulative R2Y and R2X values for the three variables were 0.97 and 0.61, respectively. The error was 0, the sensitivity was 1, the specificity was 1, and the AUC was 1.

The model separated wild gilthead sea bream from farm-raised and farm-escaped gilthead sea bream (Figure 2A) based on the intensity of the peaks in the pseudospectrum (loadings) from the PLS-LDA model (Figure 2B). The most important signals for classification were identified by the highest peaks in terms of both positive and negative values. The intense and positive signals in the pseudospectrum were higher in the group comprising farm-escaped and farm-raised fish (Figure 2A, blue circles) and corresponded mainly to creatine–creatinine, fumarate, glycine, alanine, and lactate. The negative peaks (Figure 2B) corresponded to higher intensities of taurine and TMAO in wild fish (Figure 2A, red diamonds). Although separating samples from farm-escaped and farm-raised fish was more challenging, given their similar spectra, the PLS-LDA algorithm built a model that differentiated these fish groups (Figure 3).

**Figure 4:**
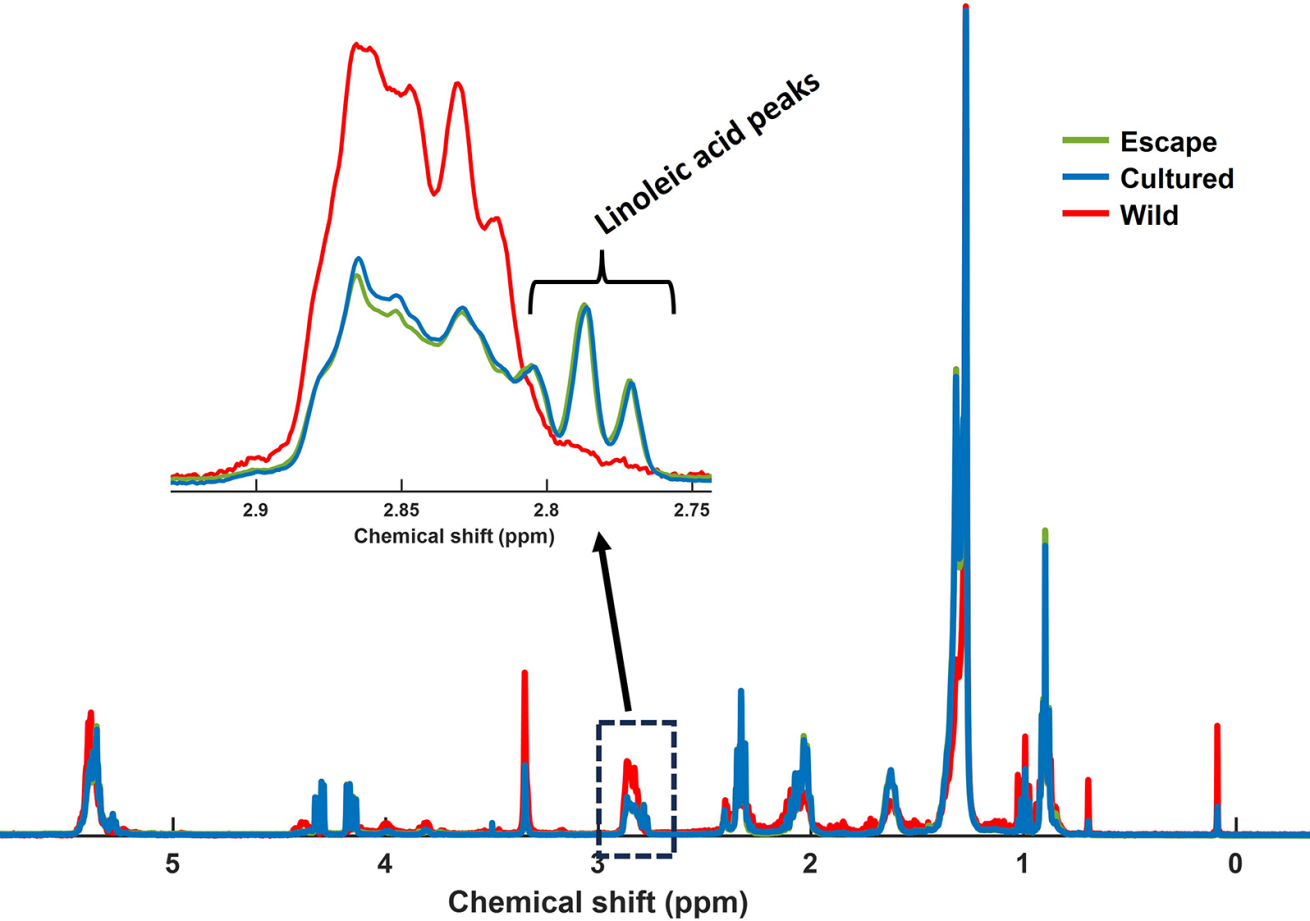
^1^H NMR spectra of the nonpolar fractions of gilthead sea bream (*Sparus aurata*) muscle samples. The inset shows the enhanced linoleic acid region.

**Figure 5:**
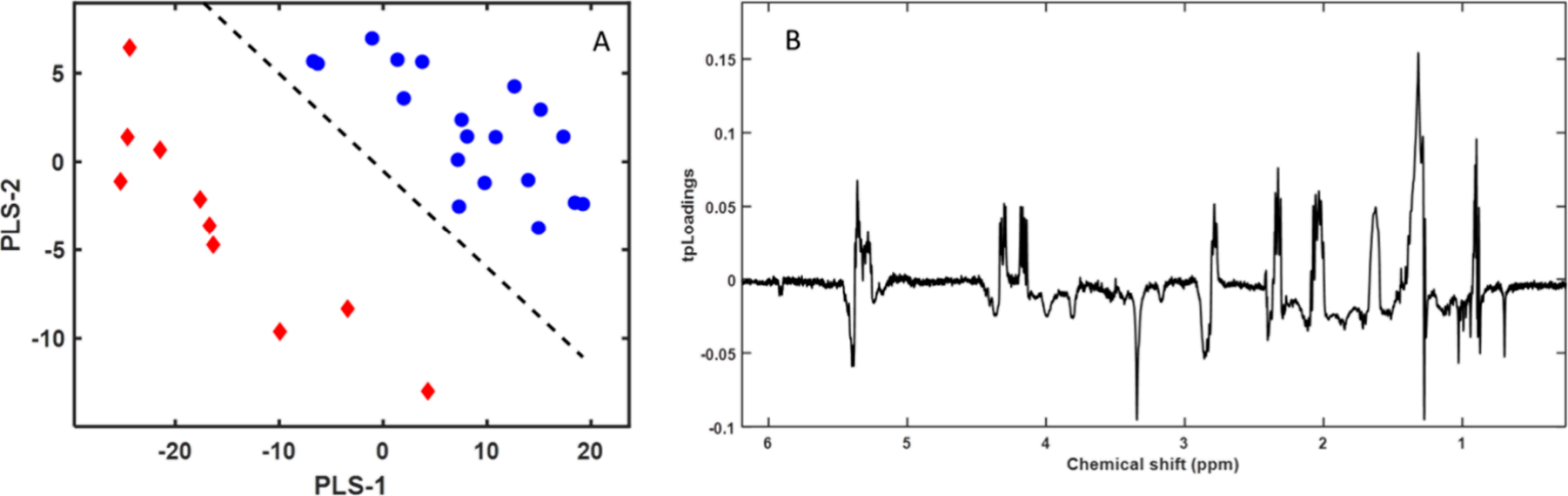
(A) First two components of the PLS-LDA model score plots of the nonpolar fraction ^1^H NMR spectra of gilthead sea bream (*Sparus aurata*) muscle samples: wild fish (red diamonds) and farm-raised and farm-escaped fish (blue circles). (B) Pseudospectrum format PLS-LDA tpLoading. Peak intensity (positive or negative) in the pseudospectrum represents the most significant spectral shift regions in the PLS-LDA model. The cumulative R2Y and R2X values for the three variables were 0.86 and 0.81, respectively. The error was 0, the sensitivity was 1, the specificity was 1, and the AUC was 1.

Figure 4 shows the nonpolar or lipid fraction spectra of the gilthead sea bream muscle samples, which had characteristics consistent with previous findings, and the signals were assigned (Table 2). Figure 5 shows the PLS-LDA models for classifying wild fish and farm-escaped or farm-raised fish. Pseudospectra (loadings) obtained from the models (Figure 5B) exhibited negative signals corresponding to abundant nonpolar compounds in wild gilthead sea bream, including higher levels of ω-3 fatty acids, whereas farm-raised and farm-escaped fish have more ω-6 fatty acids. In wild gilthead sea bream, higher levels of methyls were found, including methyls from cholesterol (0.70 ppm), methyl protons in the ω-3 polyunsaturated acyl group (0.99 ppm), methylenic protons at the position of the carbonyl group in the docosahexaenoic acyl group (2.41 ppm), bis-allylic protons in polyunsaturated fatty acids (PUFAs) (2.85 ppm), methyl protons in phosphatidylcholine (3.35 ppm), and olefinic protons in the acyl group of unsaturated fatty acid (5.39 ppm). These signals correspond to ω-3 group lipids.^29,30^

**Table 2:** Apolar Signal Assignments for ^1^H NMR Spectra of Gilthead Sea Bream (*Sparus aurata*) Muscle Samples^a^

chemical shift (ppm)	multiplicity	group	compound
0.85	m	–C**H**_3_	all acyl groups except ω-3 PUFA
0.88	t	–C**H**_3_	in fatty acyl chain
0.89	t	–C**H**_3_	unsaturated ω-6 acyl groups and FA
0.97	t	–C**H**_3_	unsaturated ω-3 acyl groups and FA
1.27	s	–(C**H**_2_)	in fatty acyl chain
1.32	s	=CHC**H**_2_CH_2_(CH_2_)–	in fatty acyl chain
1.62	m	–CO–CH_2_C**H**_2_–	in fatty acyl chain
1.63	m	–CO–CH_2_C**H**_2_–	acyl groups in 1,3-DG,1-MG and FA, except for DHA, EPA and ARA acyl groups
2.03	m	C**H**_2_CH=CH	unsaturated fatty acid
2.09	m	C**H**_2_CH=CH	unsaturated fatty acid
2.34	t	–CO–C**H**_2_–	in fatty acyl chain
2.41	m	–CO–C**H**_2_C**H**_2_–	DHA acyl groups in TG
2.77	m	=HC–C**H**_2_CH=	diunsaturated ω-6 acyl groups and FA
2.82	m	=CHC**H**_2_CH=	in fatty acyl chain
4.18	m	ROCH_2_COCOR	triglycerides
4.30	dd	ROCH_2_COCOR	triglycerides
5.35	m	–**H**C=C**H**–	in fatty acyl chain

a Abbreviations: s, singlet; d, doublet; t, triplet; m, multiplet; and dd, doublet of doublets.

Some signals were present only in farm-escaped and farm-raised gilthead sea bream. These included a triplet appearing at 2.77 ppm, corresponding to bis-allylic protons in diunited ω-6 acyl groups and fatty acids. None of the wild fish analyzed exhibited these signals, which could be due to the linoleic acid (18:2*n* – 6) present in the feed of fish raised in aquaculture.

**Figure 6:**
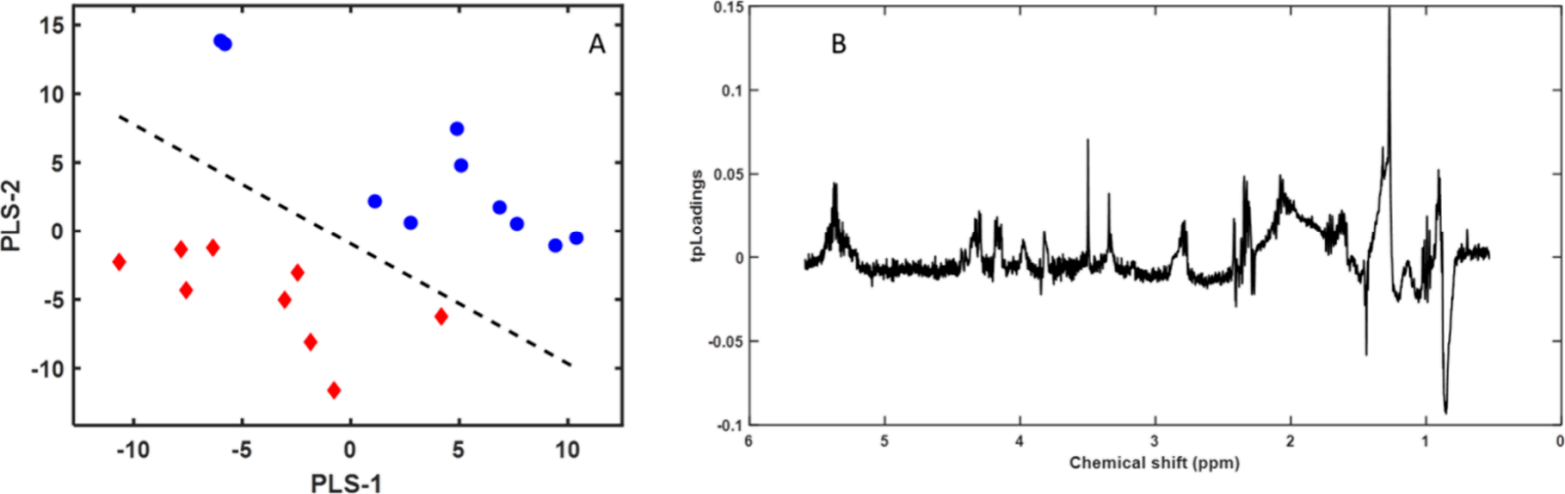
(A) First two components of the PLS-LDA model score plots of nonpolar fraction ^1^H NMR spectra of gilthead sea bream (*Sparus aurata*) muscle samples: farm-escaped fish (red diamonds) and farm-raised fish (blue circles). (B) Pseudospectrum format PLS-LDA tpLoading. Peak intensity (positive or negative) in the pseudospectrum represent the most significant spectral shift regions in the PLS-LDA model. The cumulative R2Y and R2X values for the three variables were 0.95 and 0.78, respectively. The error was 0, the sensitivity was 1, the specificity was 1, and the AUC was 1.

The model comparing the spectra of the samples of farm-escaped fish and fish reared in farms until slaughter (Figure 6) revealed differences not in the composition of ω-3 or ω-6 fatty acids but rather in the amount of free fatty acids present, as indicated by the signal at 0.89 ppm.

## 4 Discussion

The analysis of gilthead sea bream samples suggested that a possible biomarker distinguishing wild individuals from farmed individuals is the higher taurine and TMAO contents in the muscles of fish (Figure 2). Taurine is an indispensable nutrient in fish feed that must be incorporated in aquaculture feed. Although much is known about taurine metabolism, different fish species present distinct forms of taurine metabolism.^31^ Taurine plays a crucial role in various processes, such as osmoregulation, membrane stability, energy metabolism, amino acid metabolism, lipid metabolism, protein synthesis, and growth promotion.^32,33^ Therefore, taurine deficiency generates several physiological problems.^31^ Taurine supplementation has been studied in many fish species, especially carnivorous fish such as sea bream, for its critical effects on growth and amino acid and protein metabolism, which also affects lipolysis by increasing taurine levels and decreasing lipid accumulation in the muscles.^33,34^ In the present study, we identified biomarkers to differentiate wild gilthead sea bream from farmed gilthead sea bream, improving traceability without modifying the feeding of fish. However, we found that farm-raised and farm-escaped fish, which receive a taurine-supplemented diet, have markedly lower levels of taurine than their wild counterparts.

The gut microbiota plays a very important role in the production of trimethylamine oxide (TMAO).^35−39^ From dietary precursors such as choline, carnitine, betaine, and phosphatidylcholine, the microbiota produces the intermediate metabolite trimethylamine (TMA). TMA is absorbed by the circulatory system and oxidized in TMAO in a reaction catalyzed by hepatic flavin monooxygenases (FMO). TMAO may also be excreted later, although in marine organisms, it accumulates in certain tissues, such as muscle tissue. In fact, certain marine species contain large amounts of TMAO, mainly in muscle. Although it is abundant, the biological role of this Osmolite is still unclear. TMAO and other methylamine compounds are important as osmoregulators in the muscle, but this function in teleost fish is more difficult to explain, as they do not usually suffer large variations in salinity. It has also been proposed that TMAO protects proteins from high pressure at great depths, although this does not appear to be the case for sea bream, which usually live in coastal waters in the Mediterranean Sea.^40−43^

On the one hand, farmed fish have a different diet from wild fish, which could explain why the amounts of TMAO precursors present in the diet are very different for some fish and others. On the other hand, farmed fish are likely to have a different microbiota than wild fish since farmed fish are very limited to food and geographical areas and are being treated with antibiotics, which is likely to alter their microbiota, also affecting TMA production.

The higher levels of creatine and creatinine found in farm-escaped and farm-raised fish indicate the availability of fish feed as a food source.^44−46^ Creatine acts as an energy reserve, and well fed fish increase this reserve in their muscles. The enzymes responsible for creatine synthesis have been found in the muscles of different fish species at much higher levels than in the muscles of mammals, indicating the importance of creatine in amino acid metabolism in fish.^44^ The higher protein availability in farmed fish likely contributes to the higher creatine content in their muscles, relative to that in the muscles of wild fish.

Higher levels of alanine and lactate in farm-raised fish suggest that sugar metabolism (glycolysis and gluconeogenesis) is more highly weighted in these fish.^47−50^ Owing to their sedentary lifestyle, farm-raised fish have lower movement capacity and oxygen transport to tissues, which affect their aerobic and anaerobic metabolism. In contrast, wild fish have better oxygen transport to their tissues and, as a result, exhibit lower levels of anaerobic metabolism and alanine and lactate content. It has been observed that protein is the major source of energy in many fish species, with 50–70% of calories obtained from the oxidation of amino acids.^50,51^ Alanine plays a crucial role in transporting amino groups from the muscles to the liver and is synthesized from ammonium and pyruvate. Alanine, in turn, yields an amino group to alpha-ketoglutarate for the synthesis of glutamate and then urea. Pyruvate is used as a substrate for gluconeogenesis. Lactate, generated from pyruvate under conditions of high energy demand and a lack of oxygen, is rapidly oxidized back to pyruvate in the muscles. In fish, the muscles apparently function as a closed system in which lactate is not exported to the liver, as occurs in mammals.^50^

Farm-raised fish have a high-protein diet that promotes amino acid metabolism in the muscles,^50,52^ which is used as a source of energy via oxidation in the Krebs cycle, a highly active metabolic pathway in fish. High concentrations of fumarate found in the muscles of farm-escaped and farm-raised fish indicate highly active energy metabolism, as fumarate is an intermediate in the Krebs cycle. In farm-escaped fish, amino acid metabolism provides energy in the same manner, as these fish are unlikely to have easy access to wild food sources, leading to the degradation of muscle proteins for the use of amino acids as an energy source.^53^

Higher levels of glycine were also found in the muscles of farm-escaped and farm-raised fish,^54−56^ likely due to its use as a supplement in aquaculture feed.^44,51,54^ Glycine is an important amino acid for the synthesis of collagen, the main structural protein in many fish tissues,^57^ and is a precursor for creatine synthesis in the muscles. Therefore, the higher levels of glycine in farm-raised and farm-escaped fish are likely related to the higher levels of creatine observed in these fish.^51,54^ These findings suggest that glycine is another candidate biomarker for distinguishing wild gilthead sea bream from their farmed counterparts.

Amino acid metabolism in fish differs from that in other vertebrates, such as mammals. Unlike in mammals, glutamine does not play a central role in fish metabolism as a plasma nitrogen pool, which has an impact on the entire amino acid metabolism process.^51^ Amino acids serve as an important source of energy in fish and are used as carbon sources in the Krebs cycle. The content of amino acids and lipids in the diet is closely related, and the metabolism of these biomolecules determines how they are used. A high-protein intake leads to the use of proteins as an energy source and in lipogenesis, whereas an excess of lipids allows proteins to be invested in growth.^51^ The high levels of glycine found in the muscles of farm-escaped and farm-raised gilthead sea bream suggest that their metabolism is directed toward the oxidation of amino acids as a source of energy and toward lipogenesis. This is supported by the higher fat accumulation observed in farm-escaped and farm-raised fish (until slaughter) relative to that in wild fish.

The metabolic profiles of escaped farm-raised fish and farmed fish raised to slaughter were largely similar, with some notable differences. Escaped fish may have experienced feeding difficulties in the wild and may have already depleted their fat and protein reserves and glycogen stores,^53^ resulting in lower lactate levels. In contrast, farmed fish raised until slaughter may have intact protein reserves and would generate more fumarate using amino acids as an energy source. This metabolic difference could be attributed to the fasting state of the escaped fish in the wild.

The ω-3 fatty acid content in wild fish was higher than that in farm-raised fish (until slaughter) and farm-escaped fish (caught by professional fishermen), as shown in the PLS-LDA model (Figure 5), making it a useful biomarker for identifying wild fish. Fatty acids play a crucial role in energy metabolism in fish.^25,26,58−62^ They are stored as triacylglycerides in adipose tissue and are used as a continuous source of energy.^50^ As fish cannot synthesize some fatty acids, fatty acid composition is primarily determined by diet.^30^ Hence, the fatty acid profile can serve as a key biomarker for differentiating between fish groups.^63−65^ Although the diet of farm-raised fish is well controlled for optimal health, growth, and performance, it typically lacks some of the marine-originating fatty acids found in the varied diet of wild fish. As a result, farm-raised fish exhibit a lower proportion of ω-3 fatty acids and a higher proportion of ω-6 fatty acids from plant sources, including vegetable seeds.^66,67^

We found that linoleic acid (C18:2), with one of the double bonds at carbon ω-6, is present only in farm-raised and farm-escaped fish (Figure 5B), making it a valuable biomarker for identifying marine aquaculture fish. This is consistent with previous studies that have also identified linoleic acid as a key marker for the traceability of farmed fish.^68^ Therefore, the presence of this fatty acid in the muscles of farm-raised fish provides an unequivocal means of identification, ensuring their traceability.

Marine algae offer a wide range of PUFAs, including C16 (with 2–4 ethylenic bonds), C18 (with 2–5 ethylenic bonds), C20 (with 2–5 ethylenic bonds), and C22 (with 2–6 ethylenic bonds) fatty acids.^30^ Most of these PUFAs belong to the *n*– 3 family, although *n* – 6, *n* – 1, *n*– 4, and *n*– 7 PUFAs have been identified.^30,69^ Fish obtain their primary PUFAs, including α-linolenic acid (18:3*n*– 3), a metabolic precursor of 20:5*n*– 3 and 22:6*n*– 3, and linoleic acid (18:2*n*– 6), a metabolic precursor of arachidonic acid (20:4*n*– 6),^53^ from their diet because they lack Δ12 and Δ15 (ω-3) desaturases.^30^ An abundance of these fatty acids, including 20:5*n* – 3 and 22:6*n* – 3, are found in wild fish, whereas they are less abundant in farm-raised fish. These dietary differences allow us to differentiate these fish groups.^64,66^ Linoleic acid is present in farm-raised fish but not in the tissues of wild fish, making it a promising candidate biomarker for ensuring the traceability of fish reaching the market.

When comparing farm-escaped gilthead sea bream and farmed gilthead sea bream raised to slaughter, we found higher levels of free fatty acids in the muscle tissues of escaped fish. This is likely due to captive fish, finding it difficult to find food in the wild, meaning that they must mobilize fat reserves in their tissues as an energy source.^12,30^ The lipid composition of the sea bream muscle samples should be more stable over time than that of the polar metabolites and is strongly influenced by the diet of the fish. Farm-escaped fish show less variation in their lipid composition, which is similar to that of farm-reared fish kept until slaughter.

One variable that we have not considered is the duration of time spent by an escaped fish outside the cage, which may affect its ability to feed and could cause metabolic changes. The difference in metabolites between farm-escaped fish and farmed fish kept to slaughter may be due to stress induced by environmental factors, such as strong storms (which can break cages) and time spent in the wild. Lipids in the muscles are highly dependent on the diet of fish,^70^ and changes in lipid composition are slow, even with dietary changes such as those experienced by farm-escaped fish. The diet of wild fish differs from that of farmed fish, so these fish groups should exhibit different lipid profiles.^58^

In conclusion, metabolomics and lipidomics provide valuable biomarkers for tracing the origin of gilthead sea bream, differentiating wild specimens from those raised in aquaculture facilities. Taurine and linoleic acid, as well as ω-3 fatty acids, are potential biomarkers for this purpose. These tools offer a promising alternative to less effective tracing procedures.
